# Supplementary material for: Fine-Tuning of DADA2 Parameters for Multiregional Metabarcoding Analysis of 16S rRNA Genes from Activated Sludge and Comparison of Taxonomy Classification Power and Taxonomy Databases
Source: Int J Mol Sci. 2024 Mar 20;25(6):3508. doi: 10.3390/ijms25063508 (PMC10971298; doi:10.3390/ijms25063508)
Supplement: Supplementary file 1 [file ijms-25-03508-s001.zip › Table S1.pdf]

Table S1. DNA concentration (mg/μl) and date of collection of the seven samples tested.

Lp	Sampling time	Concentration mg/μl
1.	June 2021	304
2.	July 2021	300
3	October 2021	252
4.	December 2021	251
5.	February 2022	454
6.	March 2022	325
7.	May 2022	428
