# Supplementary figures and images for: Fine-Tuning of DADA2 Parameters for Multiregional Metabarcoding Analysis of 16S rRNA Genes from Activated Sludge and Comparison of Taxonomy Classification Power and Taxonomy Databases
Source: Int J Mol Sci. 2024 Mar 20;25(6):3508. doi: 10.3390/ijms25063508 (PMC10971298; doi:10.3390/ijms25063508)

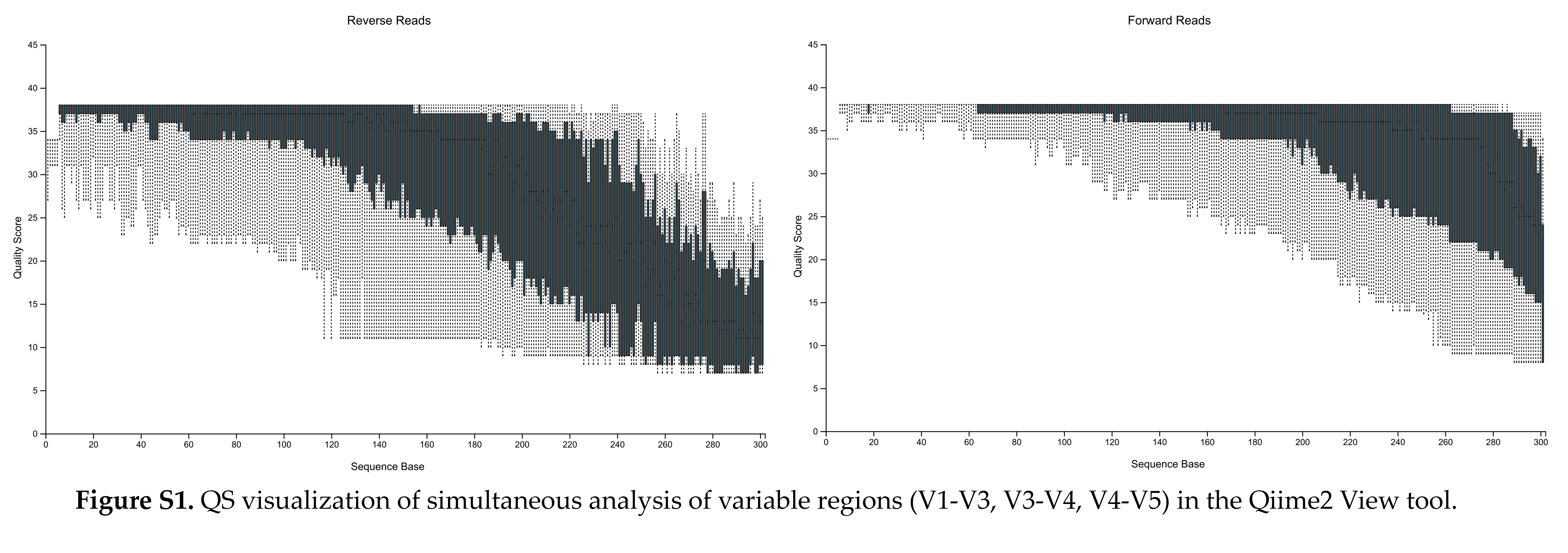

Supplement: Supplementary file 1 [file ijms-25-03508-s001.zip › Figure S1.tif]
